# Synthesis and biological evaluation of lycorine derivatives as dual inhibitors of human acetylcholinesterase and butyrylcholinesterase

**DOI:** 10.1186/1752-153X-6-96

**Published:** 2012-09-08

**Authors:** Yue-Hu Wang, Qin-Li Wan, Cheng-Ding Gu, Huai-Rong Luo, Chun-Lin Long

**Affiliations:** 1 Key Laboratory of Economic Plants and Biotechnology, Kunming Institute of Botany, Chinese Academy of Sciences, Kunming, 650201, PR China; 2 State Key Laboratory of Phytochemistry and Plant Resources in West China, Kunming Institute of Botany, Chinese Academy of Sciences, Kunming, 650201, PR China; 3 School of Chemistry and Chemical Engineering, Nanjing University, Nanjing, 210093, PR China; 4 College of Life and Environmental Sciences, Minzu University of China, Beijing, 100081, PR China

**Keywords:** Amaryllidaceae alkaloids, Lycorine, Acetylcholinesterase, Butyrylcholinesterase

## Abstract

**Background:**

Alzheimer’s disease (AD) is a neurologically degenerative disorder that affects more than 20 million people worldwide. The selective butyrylcholinesterase (BChE) inhibitors and bivalent cholinesterase (ChE) inhibitors represent new treatments for AD.

**Findings:**

A series of lycorine derivatives (**1**–**10**) were synthesized and evaluated for anti-cholinesterase activity. Result showed that the novel compound 2-*O*-*tert*-butyldimethylsilyl-1-*O*-(methylthio)methyllycorine (**7**) was a dual inhibitor of human acetylcholinesterase (hAChE) and butyrylcholinesterase (hBChE) with IC_50_ values of 11.40 ± 0.66 μM and 4.17 ± 0.29 μM, respectively. The structure-activity relationships indicated that (i) the 1-*O*-(methylthio)methyl substituent in lycorine was better than the 1-*O*-acetyl group for the inhibition of cholinesterase; (ii) the acylated or etherified derivatives of lycorine and lycorin-2-one were more potent against hBChE than hAChE; and (iii) the oxidation of lycorine at C-2 decreases the activity.

**Conclusion:**

Acylated or etherified derivatives of lycorine are potential dual inhibitors of hBChE and hAChE. Hence, further study on the modification of lycorine for ChE inhibition is necessary.

## Findings

Alzheimer’s disease (AD) is a neurologically degenerative disorder that affects more than 20 million people worldwide [1], and is the third-most costly disease after cardiovascular disease and cancer [2]. The neuropathological hallmarks of the disease include β-amyloid (Aβ) plaques, neurofibrillary tangles, and synaptic loss. Based on the cholinergic hypothesis, the symptoms of AD are the result of the reduction in brain acetylcholine (ACh) activity due to the catabolism of ACh by its principal hydrolytic enzyme acetylcholinesterase (AChE). AChE inhibition is the current approach for AD treatment. Tacrine, donepezil, rivastigmine, and galanthamine are all examples of typical AChE inhibitory drugs [3].

Similar to AChE, butyrylcholinesterase (BChE) can also inactivate ACh. The reduction in ACh is usually accompanied by a decrease in AChE activity. By contrast, BChE in AD remains at normal levels or even elevated in the brain. BChE may be a significant contributor to the observed loss of ACh in AD [4]. Furthermore, BChE inhibition can lower Aβ peptide [5,6]. BChE is essential in AD plaque maturation [7]. Selective BChE inhibition may be crucial in the mid to late stages of AD pathogenesis to circumvent further decline in mental and cognitive ability as the depletion of cholinergic neurons persists [3]. Hence, selective BChE inhibitors or bivalent ChE inhibitors represent a new treatment for AD.

**Figure 1:**
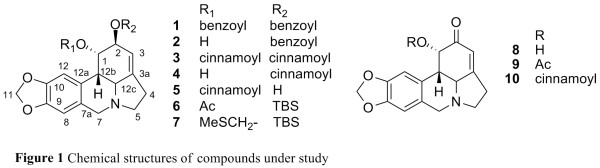
Chemical structures of compounds under study.

Lycorine, the most frequent alkaloid in Amaryllidaceae plants, has very weak inhibitory activity against electric eel acetylcholinesterase (eeAChE), with an IC_50_ value of 213 μM [8]. Acylated or etherified derivatives of lycorine, such^,^as 1-*O*-acetyllycorine and 1-*O*-acetyl-2-*Otert*-butyldimethylsilyllycorine (**6**, Figure 1), possess potent activity against eeAChE [8,9]. However, the inhibitory effect of analogues on BChE has not been reported. In our continuing work on Amaryllidaceae alkaloids [10-12], the present study reports the synthesis of lycorine derivatives (**110**), and their biological evaluation for inhibition of ChE.

Previous researchers considered that a hydrogen-bond acceptor at the C-1 of lycorine is necessary for AChE inhibitory activity, and a bulky, lipophilic substituent, such as the TBS group, at C-2 increases the activity [9,13]. Therefore, in the present study, benzoic acid or cinnamic acid were used to acylate the 1-OH and/or 2-OH of lycorine and its C-1 or C-2 oxidation derivatives. Mono- or di-acylated derivatives (**15**, and **10**) of lycorine and lycorin-2-one (**8**) were obtained by Steglich esterification (DCC/DMAP). Lycorine oxidation using pyridinium chlorochromate (PCC) in DMF yielded **8**, and the acetylated analogue (**9**) of the latter was obtained by the reaction of **8** with Ac_2_O/pyridine. The DMSO/Ac_2_O system was used to oxidate C-1 of lycorine, with the protection of 1-OH by the TBS group. However, 1-*O*-acetyl and 1-*O*-(methylthio)methyl derivatives (**6** and **7**) were obtained instead of the desired C-1 oxidation product.

The anti-ChE activity of these prepared lycorine derivatives (**110**) was evaluated by *in vitro* ChE inhibition assay, modified from Ellman’s method [14]. The results were expressed as IC_50_ values and summarized in Table 1.

**Table 1:** Inhibitory effect of compounds 1–10 on human AChE and BChE

**Lycorine analogue**	**No.**	**IC**_**50**_**(μM)**	
		**hAChE**	**hBChE**
1,2-*O*,*O*'-Dibenzoyllycorine	**1**	> 50	7.72 ± 0.26
2-*O*-Benzoyllycorine	**2**	> 50	> 50
1,2-*O*,*O*'-Di-*trans*-cinnamoyllycorine	**3**	46.76 ± 0.95	17.45 ± 0.19
2-*O*-*trans*-Cinnamoyllycorine	**4**	> 50	19.74 ± 1.37
1-*O*-*trans*-Cinnamoyllycorine	**5**	> 50	12.13 ± 0.77
1-*O*-Acetyl-2-*O*-*tert*-butyldimethylsilyllycorine	**6**	> 50	> 50
2-*O*-*tert*-Butyldimethylsilyl-1-*O*-(methylthio)methyllycorine	**7**	11.40 ± 0.66	4.17 ± 0.29
Lycorin-2-one	**8**	> 50	> 50
1-*O*-Acetyllycorin-2-one	**9**	> 50	44.46 ± 0.88
1-*O*-*trans*-Cinnamoyllycorin-2-one	**10**	> 50	20.91 ± 0.13
Tacrine (positive control)		0.26 ± 0.015	0.02 ± 0.00
Galanthamine (positive control)		1.60 ± 0.14	18.30 ± 0.14

2-*O*-*tert*-Butyldimethylsilyl-1-*O*-(methylthio)methyllycorine (**7**) showed dual inhibitory activity against both hAChE (IC_50_ = 11.40 ± 0.66 μM) and hBChE (IC_50_ = 4.17 ± 0.29 μM). The inhibitory potency of **7** was approximately four-fold stronger than that of galanthamine (IC_50_ = 18.30 ± 0.14 μM) on hBChE. Compounds **1**, and **2**−**4** also showed good effects on hBChE, with IC_50_ values of less than 20 μM.

Table 1 shows that the acylated or etherified derivatives (**1**, **3**–**5**, **7**, **9**, and **10**) of lycorine and lycorin-2-one are more potent against hBChE than hAChE. The hBChE inhibitory activity of 1-*O*-*trans*-cinnamoyllycorine (**5**, IC_50_ = 12.13 ± 0.77 μM) is about two-fold better than that of 1-*O*-*trans*-cinnamoyllycorin-2-one (**10**, IC_50_ = 20.91 ± 0.13 μM). This result implied that lycorine oxidation at C-2 may decrease the activity.

**Scheme 1:**
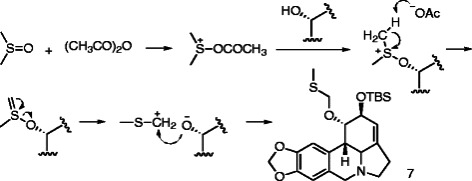
The proposed mechanism for the formation of 7.

A previous study reported that 1-*O*-acetyl-2-*Otert*-butyldimethylsilyllycorine (**6**) showed significant inhibitory activity against ACh biotransformation by eeAChE (*K*_i_ = 0.34 μM) [9]. However, in the current study, 1-*O*-acetyl-2-*Otert*-butyldimethylsilyllycorine was inactive (IC_50_ > 50 μM) against both of hAChE and hBChE. 1-*O*-(Methylthio)methyl substituent at C-1 of lycorine significantly increased the inhibitory activity against both of hAChE and hBChE in **7** compared with that of **6**. Compound **7** was an unexpected product; its formation mechanism can be explained by a Pummerer rearrangement (Scheme 1) [15].

The bioassay result of compound **7** compared with those of other tested compounds showed that a bulky, lipophilic substituent at C-1 or C-2 of lycorine is necessary for the human ChE inhibitory activity. In addition, the substituted group at C-1 is important in the activity.

The positive control tacrine showed a significant inhibitory effect on both hAChE and hBChE. However, tacrine is currently rarely used because of its hepatotoxicity [16]. Based on the results in the present study, modification of lycorine for the inhibition of ChE, especially of hBChE, is necessary.

## Experimental

### Chemistry

NMR spectra were recorded on Bruker AM-400 and DRX-500 spectrometers with TMS as an internal standard. ESIMS data were measured on an API-Qstar-Pulsar-1 instrument and HREIMS on a Waters Autospec Premier P776 mass spectrometer. Column chromatography was performed over silica gel G (80–100 and 300–400 mesh), silica gel H (10–40 μm), and Sephadex LH-20 (40–70 μm; Amersham Pharmacia Biotech AB). TLC was conducted on precoated silica gel plates GF254. HPLC separations were performed using an Agilent 1200 series pump equipped with a diode array detector, a semi-preparative Agilent Zorbax SB-C18 (5 μm, 9.4 × 250 mm) column, and a semi-preparative Waters XBridge C-18 column (5 μm, 10 × 250 mm).

#### Preparation of the acylated derivatives (1–5 and 10) of lycorine and lycorin-2-one

A suspension of lycorine or lycorin-2-one (1 mmol), cinnamic acid or benzoic acid (1 eq.), dicyclohexylcarbodiimide (1 eq.), and 4-(*N*,*N*-dimethylamino)pyridine (1 eq.) in 25 mL of dry DMF was stirred for 12 h at room temperature. The urea byproduct was filtered, and the filtrate was evaporated. The resulting residue was purified by column chromatography on silica gel, using a mixture of hexane-CHCl_3_-Me_2_CO (10:2:1), CHCl_3_, and CHCl_3_-MeOH (100:1) as eluent to yield the products (**1**–**5** and **10**).

#### 1,2-*O*,*O*'-Dibenzoyllycorine (1)

Elution with hexane-CHCl_3_-Me_2_CO (10:2:1) and CHCl_3_ afforded **1**[17] as a colorless solid, with a yield of 2.5%; ^1^H-NMR (CDCl_3_, 400 MHz): *δ* 8.06 (d, *J* = 7.7 Hz, 2H, H-2'',6''), 7.91 (d, *J* = 7.7 Hz, 2H, H-2',6'), 7.55 (m, 2H, H-4',4''), 7.42 (m, 4H, H-3',5',3'',5''), 6.84 (s, 1H, H-8), 6.59 (s, 1H, H-12), 6.16 (br s, 1H, H-1), 5.90 and 5.86 (s, 1H each, H_2_-11), 5.69 (br s, 2H, H-2,3), 4.09 and 3.62 (br s, 1H each, H_2_-7), 3.47 and 2.53 (m, 1H each, H_2_-5), 3.19 (m, 1H, H-12c), 3.06 (m, 1H, H-12b), 2.77 (m, 2H, H_2_-4); ESIMS *m/z*: 496 [M + H]^+^; HREIMS for C_30_H_25_NO_6_ [M]^+^: calcd. 495.1682; found: 495.1682.

#### 2-*O*-Benzoyllycorine (2)

Elution with CHCl_3_-MeOH (100:1) afforded **2**[17] as a colorless solid, with a yield of 12.0%; ^1^H-NMR (CDCl_3_, 400 MHz): ^1^H-NMR (CDCl_3_, 400 MHz): *δ* 8.02 (d, *J* = 7.3 Hz, 2H, H-2',6'), 7.54 (t, *J* = 7.3 Hz, 1H, H-4'), 7.40 (t, *J* = 7.3 Hz, 2H, H-3',5'), 6.80 (s, 1H, H-8), 6.58 (s, 1H, H-12), 5.87 and 5.87 (s, 1H each, H_2_-11), 5.58 (br s, 2H, H-2,3), 4.64 (br s, 1H, H-1), 4.16 and 3.55 (d, *J* = 14.2 Hz, 1H each, H_2_-7), 3.37 and 2.40 (m, 1H each, H_2_-5), 2.89 (d, *J* = 10.5 Hz, 1H, H-12b), 2.83 (d, *J* = 10.5 Hz, 1H, H-12c), 2.67 (m, 2H, H_2_-4); ^13^C-NMR (CDCl_3_, 100 MHz): *δ* 166.0 (O(*C*O)Ph), 146.5 (C-10), 146.2 × 2 (C-3a,9), 133.1 (C-4'), 130.0 and 129.8 (C-7a,1'), 129.7 × 2 (C-2',6'), 128.3 × 2 (C-3',5'), 127.3 (C-12a), 113.7 (C-3), 107.5 (C-8), 104.8 (C-12), 100.9 (C-11), 74.1 (C-1), 69.0 (C-2), 60.7 (C-12c), 57.0 (C-7), 53.7 (C-5), 41.8 (C-12b), 28.7 (C-4); ESIMS *m/z*: 392 [M + H]^+^; HREIMS for C_23_H_21_NO_5_ [M]^+^: calcd. 391.1420; found: 391.1413.

#### 1,2-*O*,*O*'-Di-*trans*-cinnamoyllycorine (3)

Elution with hexane-CHCl_3_-Me_2_CO (10:2:1) and CHCl_3_ afforded **3** as a colorless solid, with a yield of 3.5%; ^1^H-NMR (CDCl_3_, 400 MHz): *δ* 7.71 (d, *J* = 16.1 Hz, 1H, H-7''), 7.64 (d, *J* = 16.1 Hz, 1H, H-7'), 7.52 (m, 2H, H-2'',6''), 7.46 (m, 2H, H-2',6'), 7.38 (m, 3H, H-3'',4'',5''), 7.34 (m, 3H, H-3',4',5'), 6.84 (s, 1H, H-8), 6.60 (s, 1H, H-12), 6.45 (d, *J* = 16.1 Hz, 1H, H-8''), 6.32 (d, *J* = 16.1 Hz, 1H, H-8'), 5.99 (br s, 1H, H-1), 5.90 and 5.88 (s, 1H each, H_2_-11), 5.65 (br s, 1H, H-2), 5.51 (br s, 1H, H-3), 4.18 and 3.62 (d, *J* = 11.4 Hz, 1H each, H_2_-7), 3.40 and 2.50 (m, 1H each, H_2_-5), 3.06 (d, *J* = 9.6 Hz, 1H, H-12c), 2.97 (m, 1H, H-12b), 2.73 (m, 2H, H_2_-4); ^13^C-NMR (CDCl_3_, 100 MHz): *δ* 165.8 and 165.5 (C-9',9''), 146.4 (C-10), 145.8 and 145.5 × 3 (C-3a,9,7',7''), 134.3 and 134.1 (C-1',1''), 130.4 and 130.4 (C-7a,12a), 128.9 × 2 and 128.8× 2 (C-3',5',3'',5''), 128.1 × 4 (C-2',6',2'',6''), 126.6 × 2 (C-4',4''), 117.7 and 117.3 (C-8',8''), 114.3 (C-3), 107.4 (C-8), 105.2 (C-12), 101.0 (C-11), 70.7 (C-1), 69.1 (C-2), 61.3 (C-12c), 56.7 (C-7), 53.7 (C-5), 40.4 (C-12b), 28.8 (C-4); ESIMS *m/z*: 548 [M + H]^+^; HREIMS for C_34_H_29_NO_6_ [M]^+^: calcd. 547.1995; found: 547.1999.

#### 2-*O*-*trans*-Cinnamoyllycorine (4)

Elution with CHCl_3_-MeOH (100:1) afforded **4** as a colorless solid, with a yield of 16.7%; ^1^H-NMR (CDCl_3_, 400 MHz): *δ* 7.70 (d, *J* = 16.1 Hz, 1H, H-7'), 7.50 (m, 2H, H-2',6'), 7.38 (m, 3H, H-3',4',5'), 6.84 (s, 1H, H-8), 6.61 (s, 1H, H-12), 6.43 (d, *J* = 16.1 Hz, 1H, H-8'), 5.91 and 5.90 (s, 1H each, H_2_-11), 5.57 (br s, 1H, H-2), 5.48 (br s, 1H, H-3), 4.62 (br s, 1H, H-1), 4.14 and 3.68 (d, *J* = 14.1 Hz, 1H each, H_2_-7), 3.38 and 2.59 (m, 1H each, H_2_-5), 3.01 (m, 1H, H-12b), 2.82 (d, *J* = 8.7 Hz, 1H, H-12c), 2.71 (m, 2H, H_2_-4); ^13^C-NMR (CDCl_3_, 100 MHz): *δ* 166.3 (C-9'), 146.4 (C-10), 145.5 × 3 (C-3a,9,7'), 134.2 (C-1'), 130.4 × 2 (C-7a,12a), 128.9 × 2 (C-3',5'), 128.1 × 2 (C-2',6'), 127.3 (C-4'), 117.7 (C-8'), 113.8 (C-3), 107.6 (C-8), 104.8 (C-12), 101.0 (C-11), 73.5 (C-1), 68.9 (C-2), 60.5 (C-12c), 56.5 (C-7), 53.8 (C-5), 41.3 (C-12b), 28.9 (C-4); ESIMS *m/z*: 418 [M + H]^+^; HREIMS for C_25_H_23_NO_5_ [M]^+^: calcd. 417.1576; found: 417.1567.

#### 1-*O*-*trans*-Cinnamoyllycorine (5)

Elution with CHCl_3_-MeOH (50:1) afforded **5** as a colorless solid, with a yield of 1.4%; ^1^H-NMR (CDCl_3_, 400 MHz): ^1^H-NMR (CDCl_3_, 400 MHz): *δ* 7.59 (d, *J* = 16.0 Hz, 1H, H-7'), 7.45 (m, 2H, H-2',6'), 7.33 (m, 3H, H-3',4',5'), 6.64 (s, 1H, H-8), 6.59 (s, 1H, H-12), 6.28 (d, *J* = 16.0 Hz, 1H, H-8'), 5.89 and 5.87 (s, 1H each, H_2_-11), 5.72 (br s, 1H, H-1), 5.59 (br s, 1H, H-3), 4.27 (br s, 1H, H-2), 4.19 and 3.61 (d, *J* = 14.0 Hz, 1H each, H_2_-7), 3.37 and 2.51 (m, 1H each, H_2_-5), 2.95 (m, 2H, H-12b,12c), 2.67 (m, 2H, H_2_-4); ^13^C-NMR (CDCl_3_, 100 MHz): *δ* 166.6 (C-9'), 146.6 (C-10), 146.3 and 145.5 × 2 (C-3a,9,7'), 134.1 (C-1'), 130.4 × 2 (C-7a,12a), 128.8 × 2 (C-3',5'), 128.1 × 2 (C-2',6''), 127.1 (C-4'), 117.5 (C-8'), 113.8 (C-3), 107.4 (C-8), 104.9 (C-12), 100.9 (C-11), 72.6 (C-1), 69.4 (C-2), 61.5 (C-12c), 56.8 (C-7), 53.7 (C-5), 39.1 (C-12b), 28.7 (C-4); ESIMS *m/z*: 418 [M + H]^+^; HREIMS for C_25_H_23_NO_5_ [M]^+^: calcd. 417.1576; found: 417.1583.

#### 1-*O*-*trans*-Cinnamoyllycorin-2-one (10)

Elution with hexane-CHCl_3_-Me_2_CO (10:2:1), CHCl_3_, and CHCl_3_-MeOH (100:1) afforded **10** as a colorless solid, with a yield of 62.7%; ^1^H-NMR (CDCl_3_, 400 MHz): *δ* 7.63 (d, *J* = 16.0 Hz, 1H, H-7'), 7.44 (m, 2H, H-2',6'), 7.34 (m, 3H, H-3',4',5'), 6.79 (s, 1H, H-8), 6.57 (s, 1H, H-12), 6.28 (d, *J* = 16.0 Hz, 1H, H-8'), 6.14 (d, *J* = 1.6 Hz, 1H, H-1), 6.03 (br s, 1H, H-3), 5.90 and 5.89 (s, 1H each, H_2_-11), 4.18 and 3.65 (d, *J* = 14.0 Hz, 1H each, H_2_-7), 3.48 and 2.59 (m, 1H each, H_2_-5), 3.35 (m, 2H, H-12b,12c), 2.90 (m, 2H, H_2_-4); ^13^C-NMR (CDCl_3_, 100 MHz): *δ* 204.4 (C-2), 165.5 (C-9'), 146.7 × 2 (C-3a,10), 146.1 × 2 (C-9,7'), 134.1 (C-1'), 130.5 × 2 (C-7a,12a), 128.8 × 2 (C-3',5'), 128.1 × 2 (C-2',6''), 125.2 (C-4'), 120.6 (C-3), 116.9 (C-8'), 107.3 (C-8), 105.4 (C-12), 101.1 (C-11), 68.9 (C-1), 62.3 (C-12c), 56.1 (C-7), 53.2 (C-5), 45.4 (C-12b), 30.0 (C-4); ESIMS *m/z*: 416 [M + H]^+^; HREIMS for C_25_H_21_NO_5_ [M]^+^: calcd. 415.1420; found: 415.1418.

#### Synthesis of 1-*O*-acetyl-2-*O*-*tert*-butyldimethylsilyllycorine (6) and 2-*O*-*tert*-butyldimethylsilyl-1-*O*-(methylthio)methyllycorine (7)

A solution of 2-*Otert*-butyldimethylsilyllycorine [9] (60 mg, 0.150 mmol), dry dimethyl sulfoxide (0.26 mL), and acetic anhydride (0.18 mL) was stirred overnight at room temperature. Afterward, the reaction mixture was quenched with H_2_O (0.7 mL) and aqueous NH_4_OH (0.4 mL). The resulting solution was extracted with Et_2_O. The organic layer was separated, dried over Na_2_SO_4_, and then concentrated. The residue was purified by silica gel column chromatography (petrol/EtOAc, 10:1) and HPLC with a Waters XBridge C-18 column (5 μm, 10 × 250 mm) using MeOH-H_2_O (95:5) as eluent to yield **6**[9] (*t*_R_ = 9.876 min, 23 mg, 0.0530 mmol) and **7** (*t*_R_ = 11.955 min, 17 mg, 0.0368 mmol).

#### 1-*O*-Acetyl-2-*O*-*tert*-butyldimethylsilyllycorine (6)

A white solid, yield 35.4%; ^1^H-NMR (CDCl_3_, 400 MHz): *δ* 6.73 (s, 1H, H-8), 6.56 (s, 1H, H-12), 5.92 and 5.91 (s, 1H each, H_2_-11), 5.55 (br s, 1H, H-1), 5.39 (br s, 1H, H-3), 4.17 (br s, 1H, H-2), 4.14 and 3.52 (d, *J* = 14.1 Hz, 1H each, H_2_-7), 3.36 and 2.37 (m, 1H each, H_2_-5), 2.94 (d, *J* = 8.8 Hz, 1H, H-12b), 2.74 (d, *J* = 8.8 Hz, 1H, H-12c), 2.63 (m, 2H, H_2_-4), 1.94 (s, 3H, O(CO)C*H*_3_), 0.89 (s, 9H, SiC(C*H*_3_)_3_), 0.19 and 0.11 (s, 3H each, Si(C*H*_3_)_2_); ESIMS *m/z*: 444 [M + H]^+^; HREIMS for C_24_H_33_NO_5_Si [M]^+^: calcd. 443.2128; found: 443.2127.

#### 2-*O*-*tert*-Butyldimethylsilyl-1-*O*-(methylthio)methyllycorine (7)

A pale yellow solid, yield 24.5%; ^1^H-NMR (CDCl_3_, 400 MHz): *δ* 7.02 (s, 1H, H-8), 6.56 (s, 1H, H-12), 5.91 and 5.91 (s, 1H each, H_2_-11), 5.42 (br s, 1H, H-3), 4.66 and 4.62 (d, *J* = 12.0 Hz, 1H each, OC*H*_2_SCH_3_), 4.51 (br s, 1H, H-1), 4.34 (br s, 1H, H-2), 4.12 and 3.50 (d, *J* = 14.0 Hz, 1H each, H_2_-7), 3.34 and 2.33 (m, 1H each, H_2_-5), 2.88 (d, *J* = 10.4 Hz, 1H, H-12b), 2.73 (d, *J* = 10.4 Hz, 1H, H-12c), 2.61 (m, 2H, H_2_-4), 1.97 (s, 3H, SC*H*_3_), 0.89 (s, 9H, SiC(C*H*_3_)_3_), 0.16 and 0.12 (s, 3H each, Si(C*H*_3_)_2_); ESIMS *m/z*: 462 [M + H]^+^; HREIMS for C_24_H_35_NO_4_SiS [M]^+^: calcd. 461.2056; found: 461.2058.

#### Lycorin-2-one (8)

Lycorine [10,12] (1 g, 3.481 mmol), PCC (6.657 g, 30.886 mmol) and silica gel (6.657 g) in anhydrous DMF (50 mL) were stirred at room temperature for 24 h. Afterward, the reaction mixture was filtrated through a pad of Celite. The filtrate was then poured into water, adjusted the pH to 9 using ammonia, and extracted with CHCl_3_. The solvent was evaporated under reduced pressure. The residue was purified by silica gel column chromatography (CHCl_3_-MeOH, 20:1) to yield **8**[18,19] (70 mg, 0.245 mmol).

A gray powder, yield 7.0%; ^1^H-NMR (CDCl_3_, 500 MHz): *δ* 6.77 (s, 1H, H-8), 6.60 (s, 1H, H-12), 5.97 and 5.95 (s, 1H each, H_2_-11), 5.93 (br s, 1H, H-3), 4.55 (d, *J* = 2.3 Hz, 1H, H-1), 4.16 and 3.64 (d, *J* = 14.0 Hz, 1H each, H_2_-7), 3.45 and 2.53 (m, 1H each, H_2_-5), 3.25 (br s, 1H, H-12b), 3.14 (d, *J* = 9.4 Hz, 1H, H-12c), 2.86 (br s, 2H, H_2_-4); ESIMS *m/z*: 286 [M + H]^+^; HREIMS for C_16_H_15_NO_4_ [M]^+^: calcd. 285.1001; found: 285.1000.

#### 1-*O*-Acetyllycorin-2-one (9)

A suspension of (20 mg, 0.0702 mmol) of lycorin-2-one (**8**) in 0.5 mL of pyridine and 0.5 mL Ac_2_O was stirred for 12 h at room temperature and then 20 mL of water was added. The solution was adjusted to pH 9 using ammonia (5 mL) and extracted with CHCl_3_ for four times before the removal of CHCl_3_. The resulting residue was purified by prep. TLC (CHCl_3_-MeOH, 30:1) to yield **9** (5 mg, 0.0153 mmol).

A gray solid, yield 21.8%; ^1^H-NMR (CDCl_3_, 400 MHz): *δ* 6.72 (s, 1H, H-8), 6.57 (s, 1H, H-12), 6.00 and 5.99 (s, 1H each, H_2_-11), 5.93 and 5.92 (br s, 1H each, H-1,3), 4.17 and 3.61 (d, *J* = 14.1 Hz, 1H each, H_2_-7), 3.48 and 2.52 (m, 1H each, H_2_-5), 3.26 (br d, *J* = 10.0 Hz, 1H, H-12b), 3.16 (d, *J* = 10.0 Hz, 1H, H-12c), 2.86 (m, 2H, H_2_-4), 1.96 (s, 3H, O(CO)C*H*_3_); ESIMS *m/z*: 328 [M + H]^+^; HREIMS for C_18_H_17_NO_5_ [M]^+^: calcd. 327.1107; found: 327.1105. The NMR spectra of compounds **1**–**10** were also available as a PDF file (Additional file 1).

### Cholinesterase inhibitory activity

AChE/BChE inhibitory activity of compounds **110** (purity >95%) was assayed using the spectrophotometric method developed by Ellman et al. [14], with slight modification. *S*-Acetylthiocholine iodide, *S*-butyrylthiocholine iodide, 5,5'-dithio-bis-(2-nitrobenzoic) acid (DTNB, Ellman’s reagent), hAChE, and hBChE, were purchased from Sigma Chemical. The test compounds were dissolved in DMSO. The reaction mixture contained 110 μL of phosphate buffer (pH 8.0), 10 μL of test compounds (50 μM), and 40 μL of hAChE or hBChE (0.04 U/100 μL), and the mixture was incubated for 20 min (30 °C). Subsequently, the reaction was initiated by the addition of 20 μL of DTNB (6.25 mM) and 20 μL of ACh or butyrylthiocholine (BCh) for hAChE or hBChE inhibitory activity, respectively. Hydrolysis of ACh or BCh was monitored at 405 nm after 30 min. All reactions were performed in triplicate. Inhibition percentage was calculated as follows: % inhibition = (*E* − *S*)/*E* × 100, where *E* is the enzyme activity without the test compounds and *S* is the enzyme activity with the test compounds. Inhibition curves were obtained for each compound by plotting the inhibition percentage versus the logarithm of the inhibitor concentration in the assay solution. Linear regression parameters were determined for each curve, and the IC_50_ values were extrapolated. The same procedure was applied for the positive control tacrine (Sigma, purity 98%) and galanthamine (purity >95%) [12]. The study was approved by the ethical committee in Kunming Institute of Botany (reference number 1205) and performed according to the Helsinki Declaration.

## Conclusion

A series of lycorine derivatives (**1**–**10**) were synthesized and evaluated for anti-cholinesterase activity. The novel compound 2-*O*-*tert*-butyldimethylsilyl-1-*O*-(methylthio)methyllycorine (**7**) was a dual hAChE and hBChE inhibitor. The structure-activity relationships indicated that (i) the 1-*O*-(methylthio)methyl substituent in lycorine is better than the 1-*O*-acetyl group for the inhibition of cholinesterase; (ii) the acylated or etherified derivatives of lycorine and lycorin-2-one are more potent against hBChE than hAChE; and (iii) the oxidation of lycorine at C-2 decreases the activity. Hence, further study on the modification of lycorine for the inhibition of ChE is necessary.

## Abbreviations

Aβ: *β*-amyloid; Ach: Acetylcholine; AChE: Acetylcholinesterase; Ac_2_O: Acetic anhydride; AD: Alzheimer’s disease; BChE: Butyrylcholinesterase; ChE: Cholinesterase; DMAP: 4-(*N*,*N*-dimethylamino)pyridine; DMF: *N*,*N*-dimethylformamide; DTNB: 5,5'-dithio-bis-(2-nitrobenzoic) acid; DMSO: Dimethylsulphoxide; DCC: Dicyclohexylcarbodiimide; eeAChE: Electric eel acetylcholinesterase; ESIMS: Electrospray ionization mass spectrometry; EtOAc: Ethyl acetate; hAChE: Human acetylcholinesterase; hBChE: Human butyrylcholinesterase; HPLC: High pressure liquid chromatography; HREIMS: High resolution electrospray ionization mass spectrometry; IC_50_: Concentration producing 50% inhibition; Me_2_CO: Acetone; NMR: Nuclear magnetic resonance; PCC: Pyridinium chlorochromate; TBS: *Tert*-butyldimethylsilyl; TLC: Thin layer chromatography; TMS: Tetramethylsilane.

## Competing interests

The authors declare that they have no competing interests.

## Authors’ contributions

YHW and CLL directed the whole study of the paper. The synthetic experiments were carried out by YHW and CDG. The bioassay was performed by QLW and HRL. YHW drafted the manuscript and CLL revised it. All authors read and approved the final manuscript.

## Supplementary Material

Additional file 1 NMR spectra of compounds 1–10. Click here for file
