# Supplementary material for: Synthesis and biological evaluation of lycorine derivatives as dual inhibitors of human acetylcholinesterase and butyrylcholinesterase
Source: Chem Cent J. 2012 Sep 8;6:96. doi: 10.1186/1752-153X-6-96 (PMC3782361; doi:10.1186/1752-153X-6-96)

Additional material

Contents:

A1 ^{1}H-NMR spectrum of **1**
A2 ^{1}H-NMR spectrum of **2**
A3 ^{13}C-NMR spectrum of **2**
A4 ^{1}H-NMR spectrum of **3**
A5 ^{13}C-NMR spectrum of **3**
A6 ^{1}H-NMR spectrum of **4**
A7 ^{13}C-NMR spectrum of **4**
A8 ^{1}H-NMR spectrum of **5**
A9 ^{13}C-NMR spectrum of **5**
A10 ^{1}H-NMR spectrum of **6**
A11 ^{1}H-NMR spectrum of **7**
A12 ^{1}H-NMR spectrum of **8**
A13 ^{1}H-NMR spectrum of **9**
A14 ^{1}H-NMR spectrum of **10**
A15 ^{13}C-NMR spectrum of **10**

A1 ^{1}H-NMR spectrum of **1**

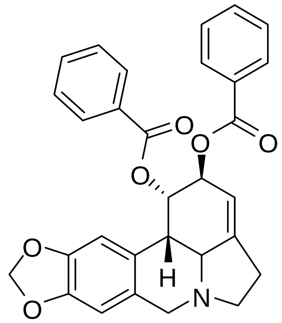

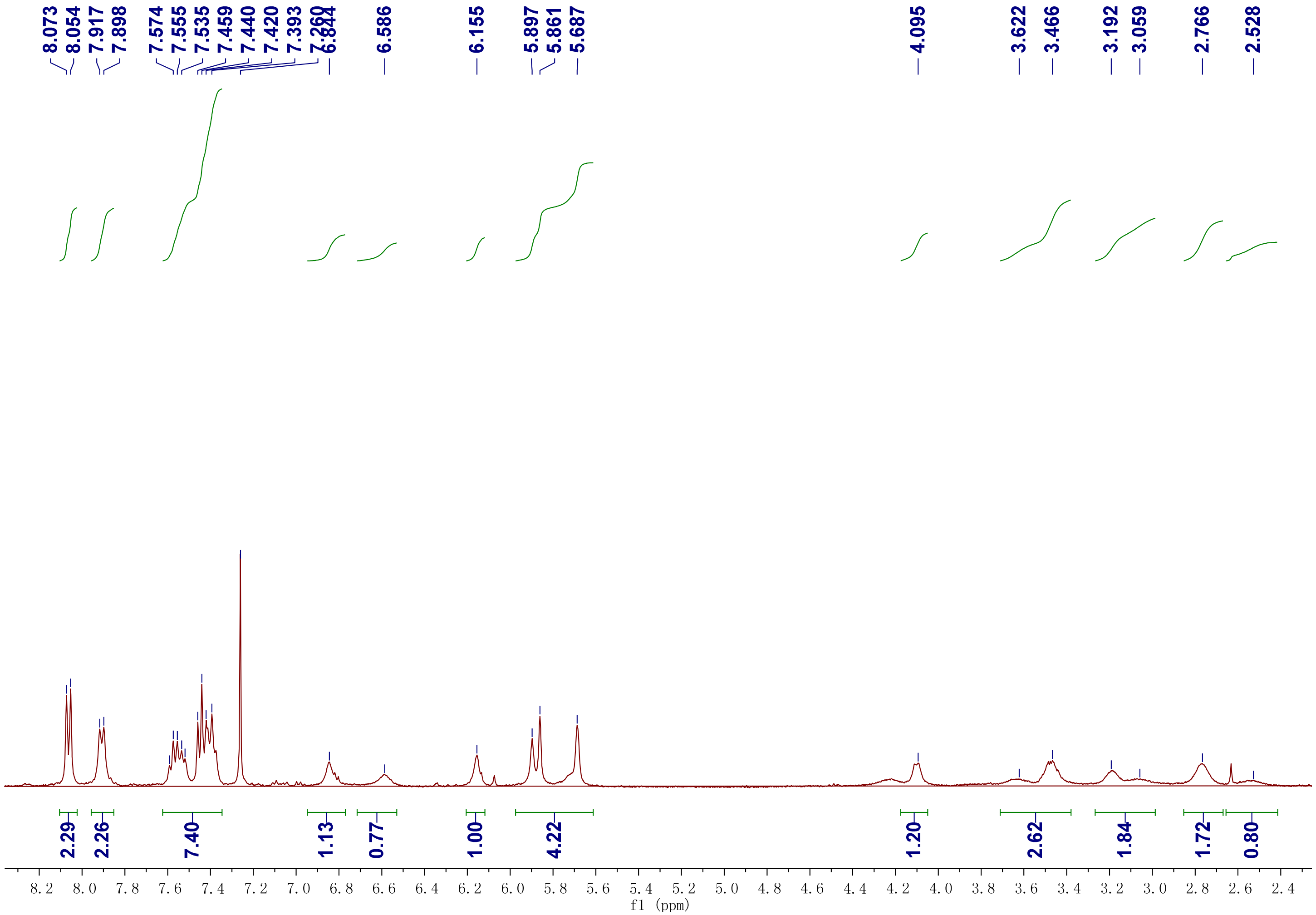

A2 ^{1}H-NMR spectrum of **2**

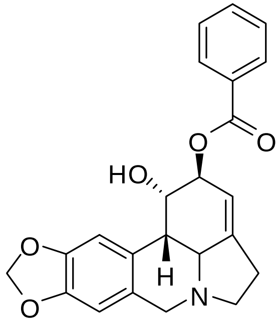

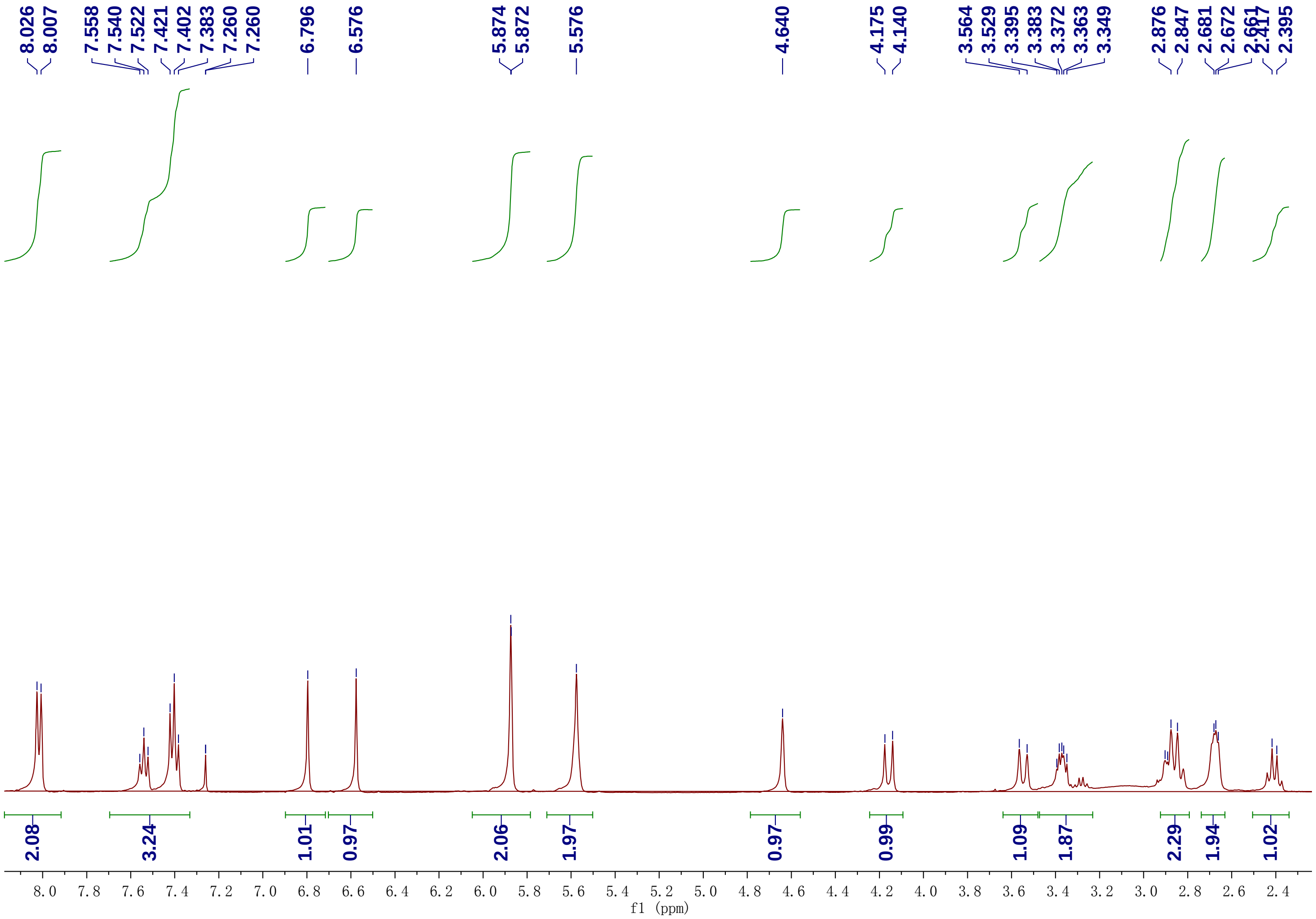

A3 ^{13}C-NMR spectrum of **2**

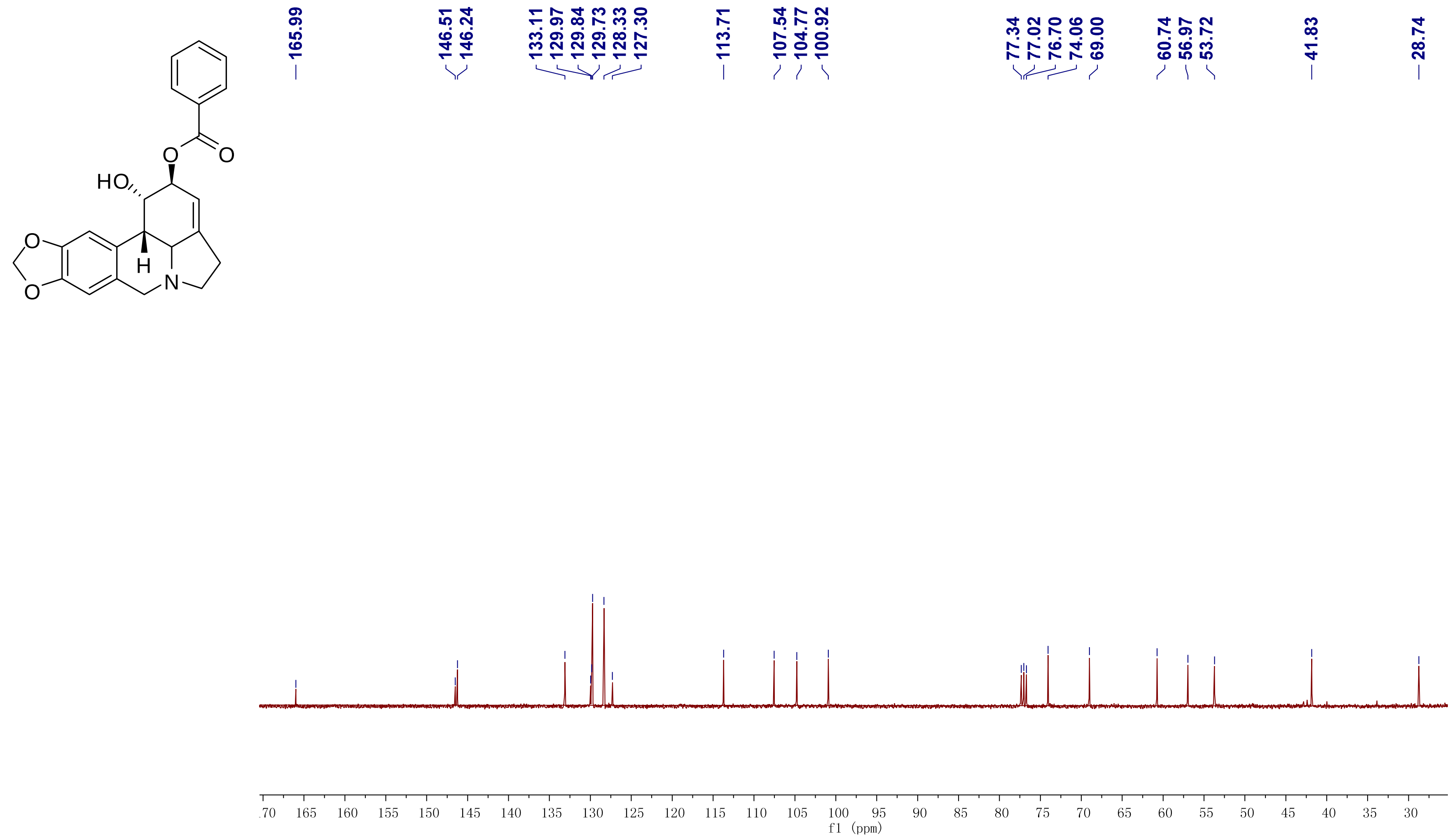

A4 ^{1}H-NMR spectrum of **3**

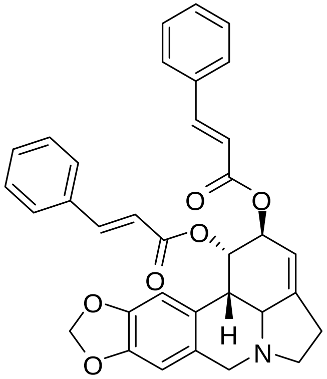

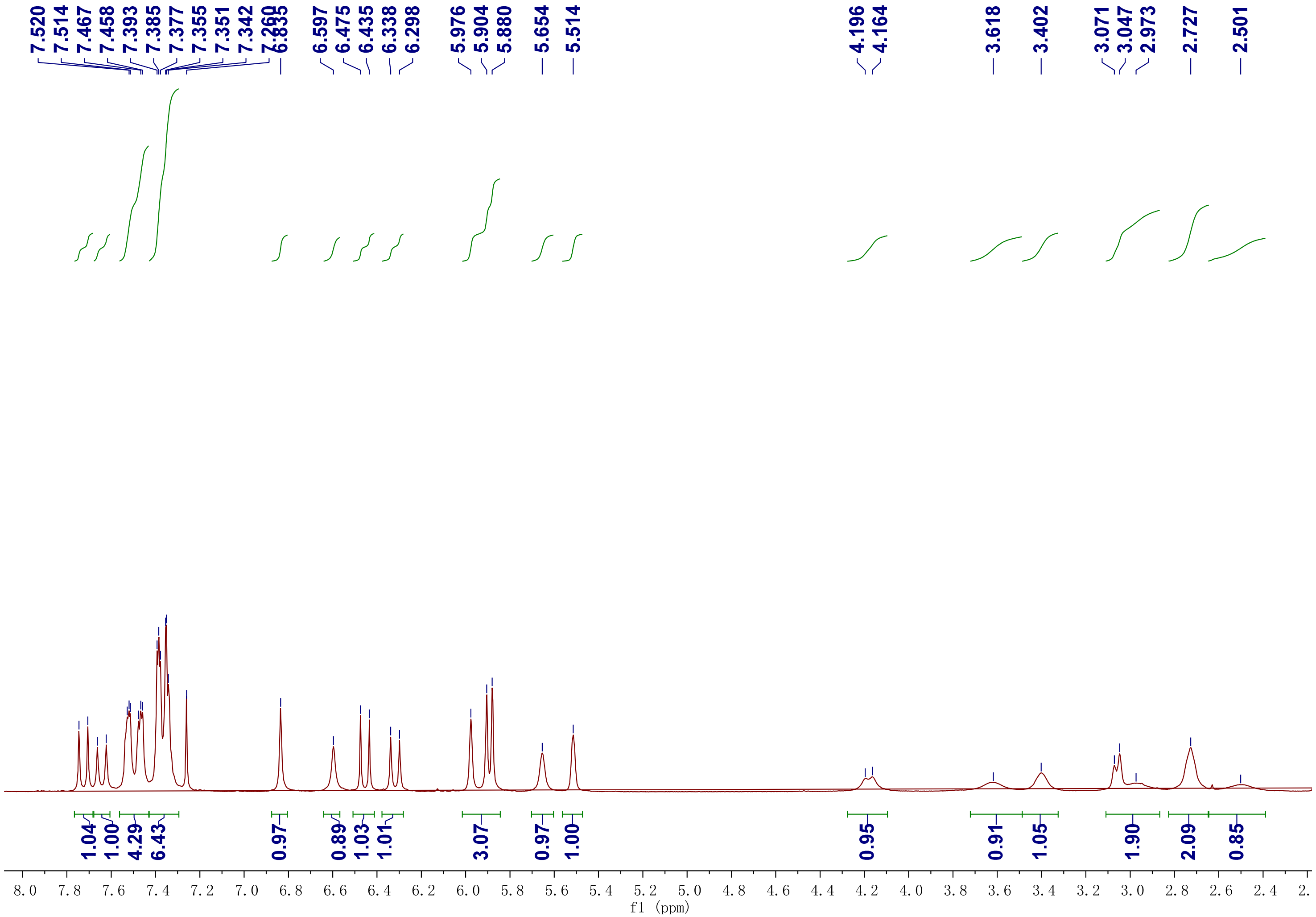

A5 ^{13}C-NMR spectrum of **3**

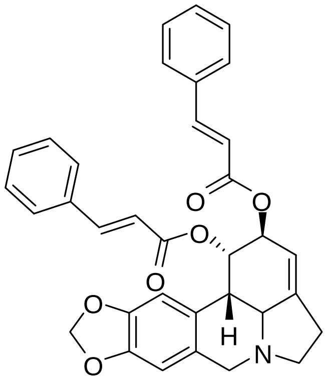

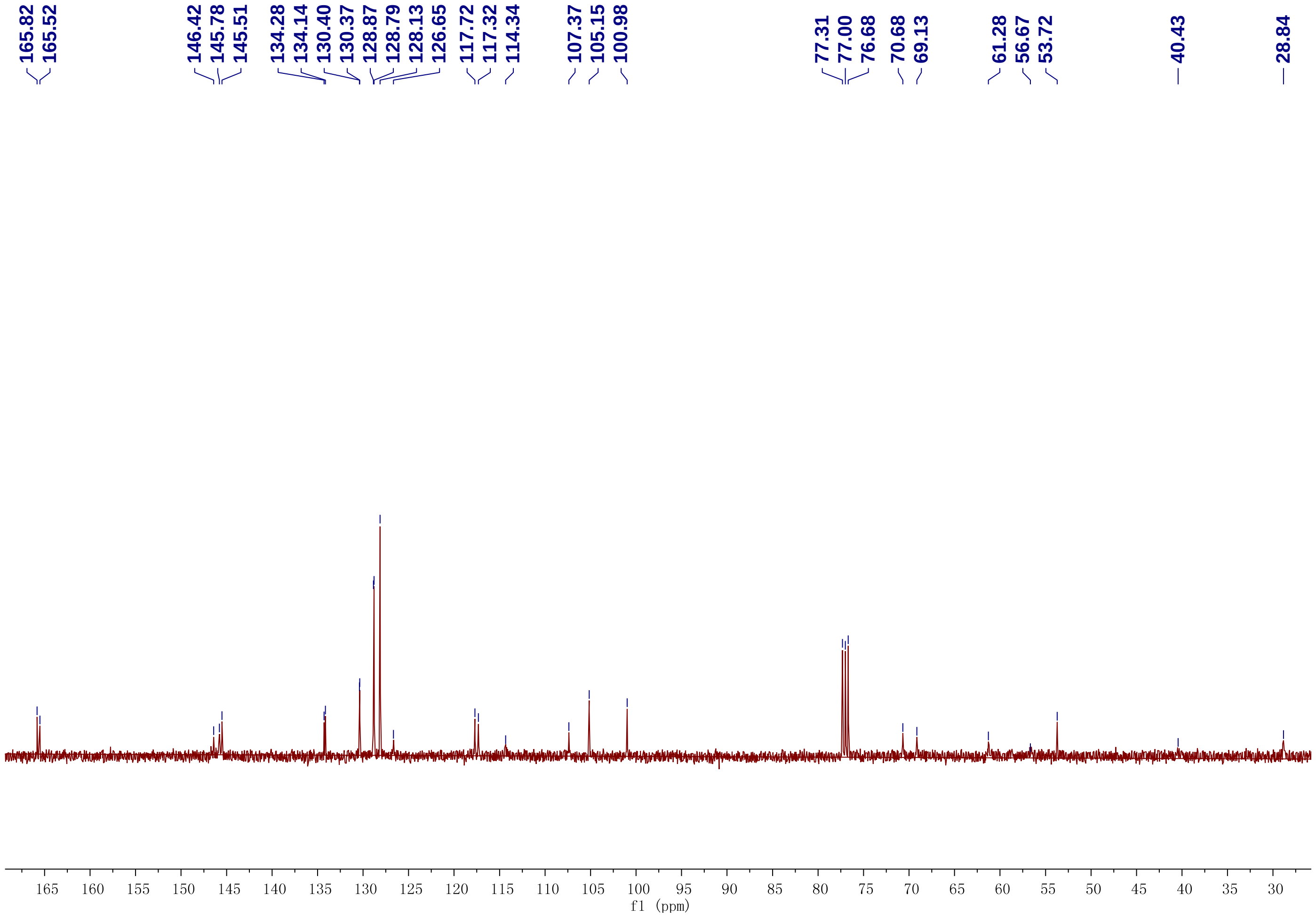

A6 ^{1}H-NMR spectrum of **4**

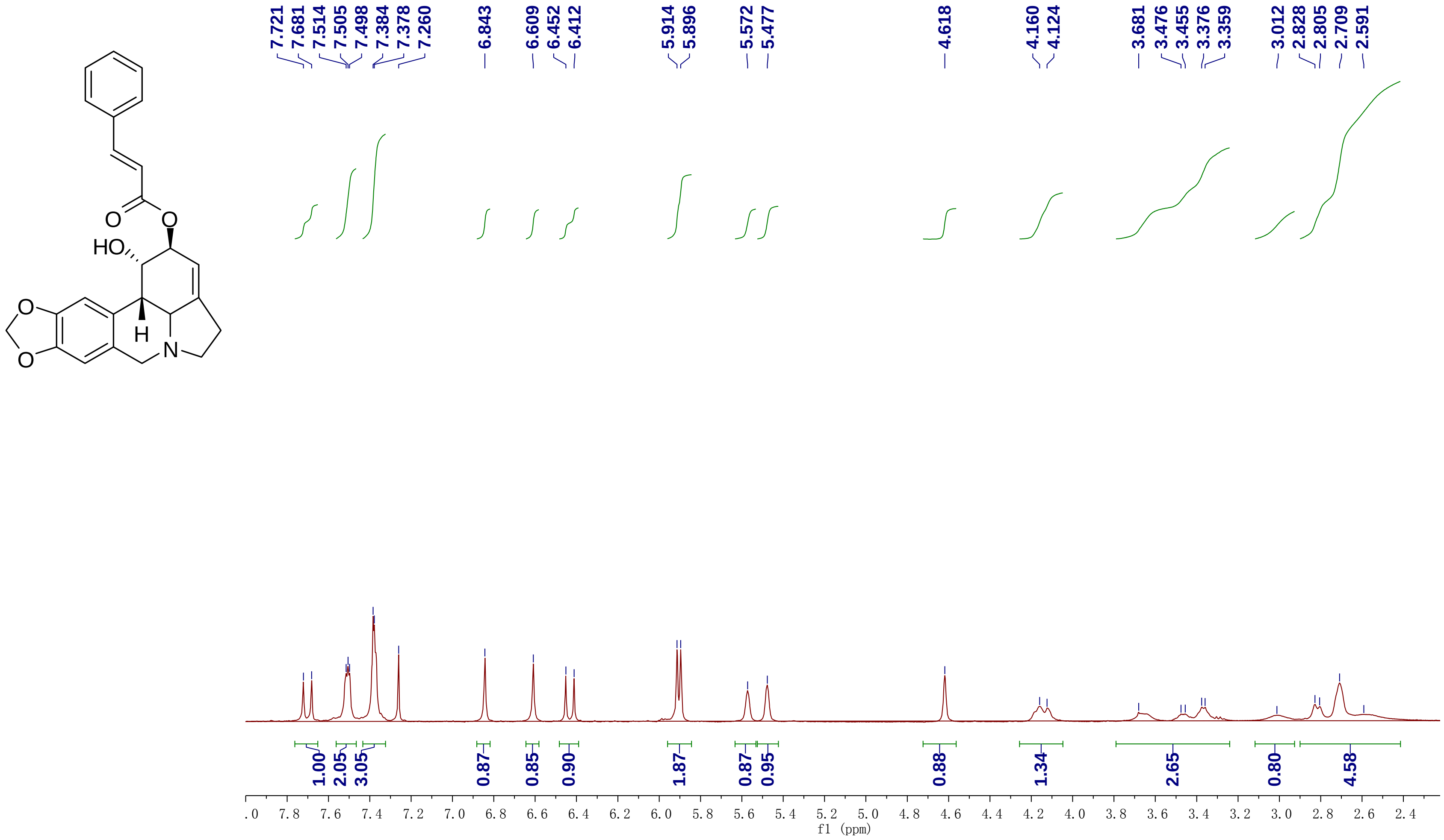

A7 ^{13}C-NMR spectrum of **4**

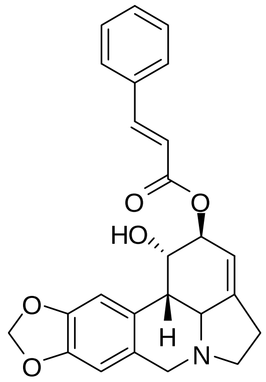

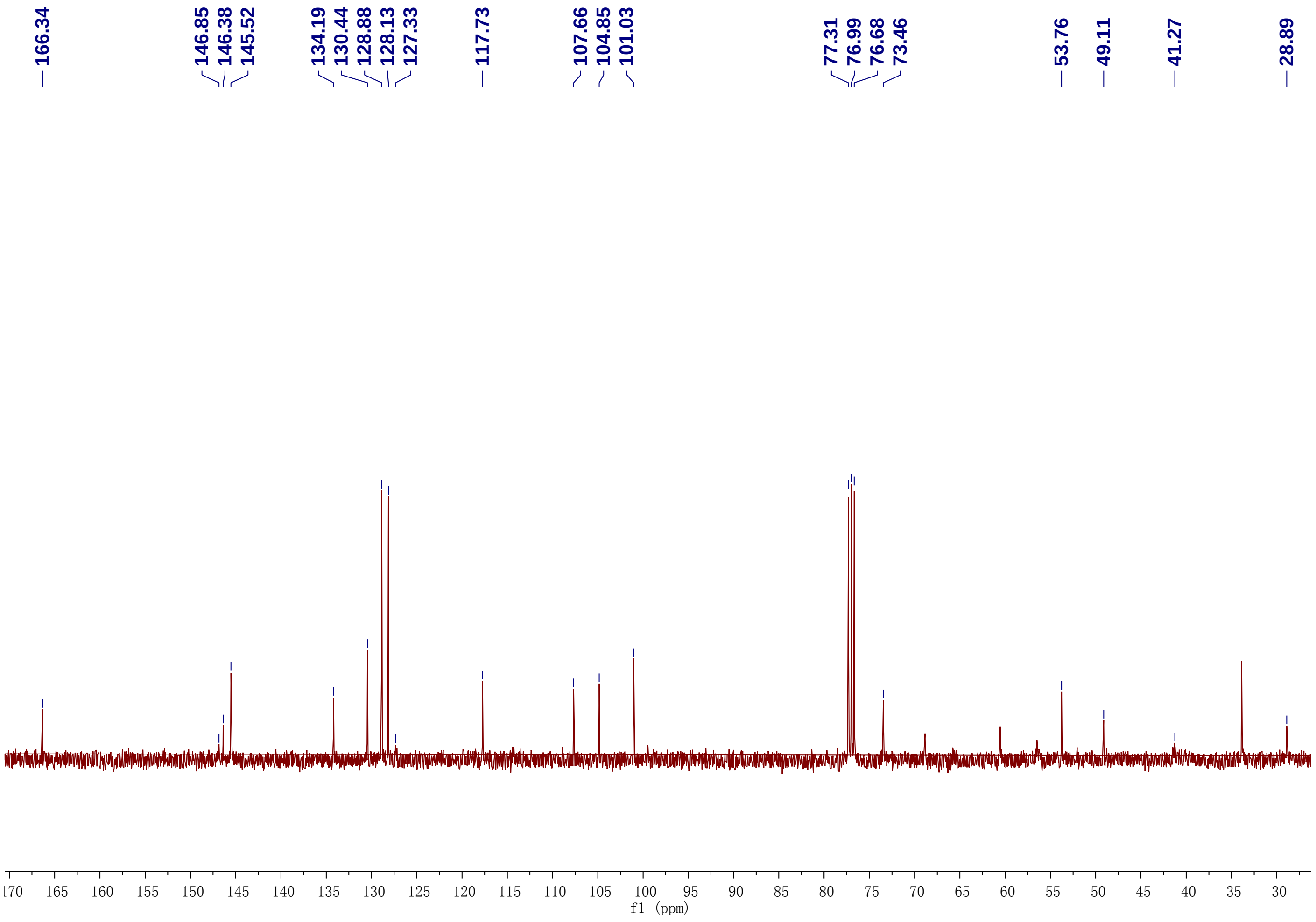

A8 ^{1}H-NMR spectrum of **5**

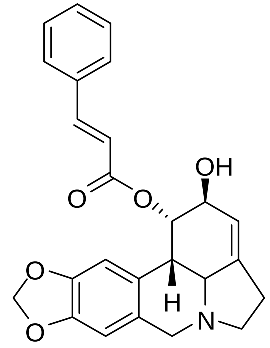

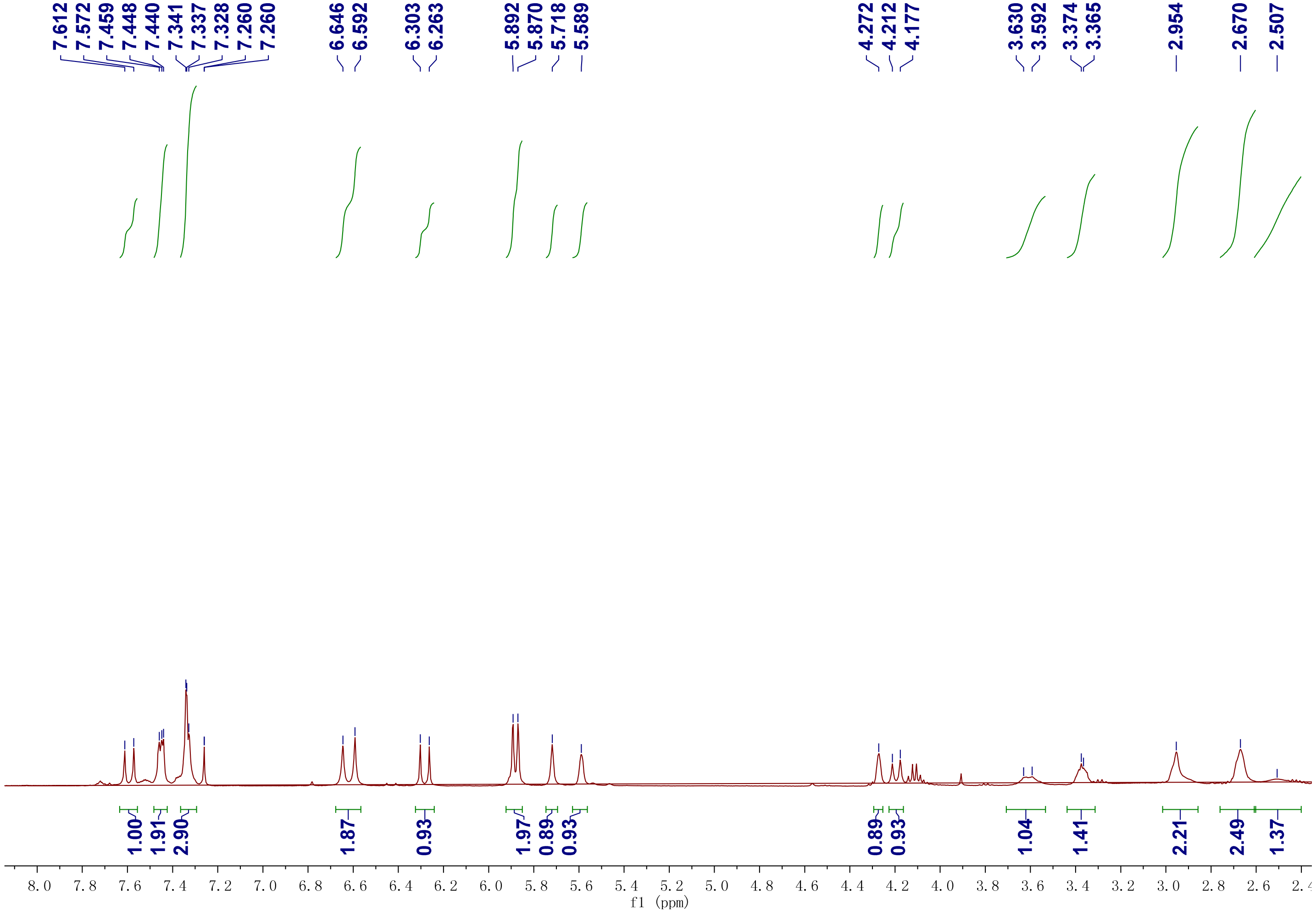

A9 ^{13}C-NMR spectrum of **5**

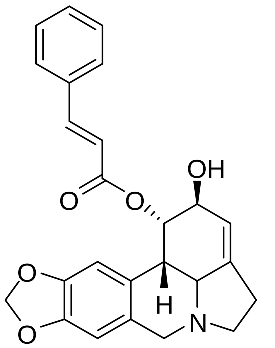

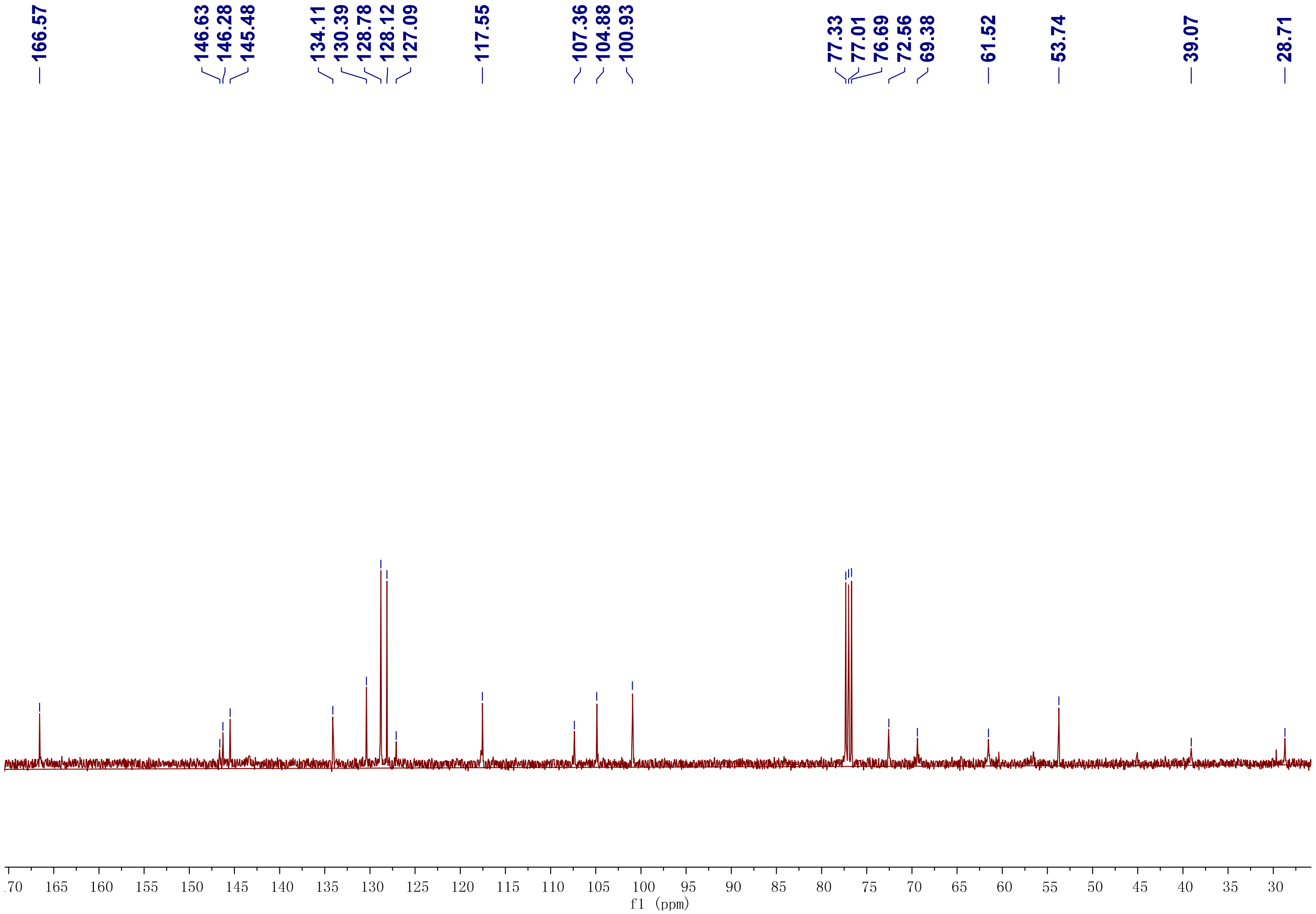

A10 ^{1}H-NMR spectrum of **6**

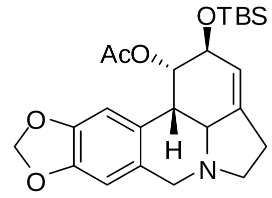

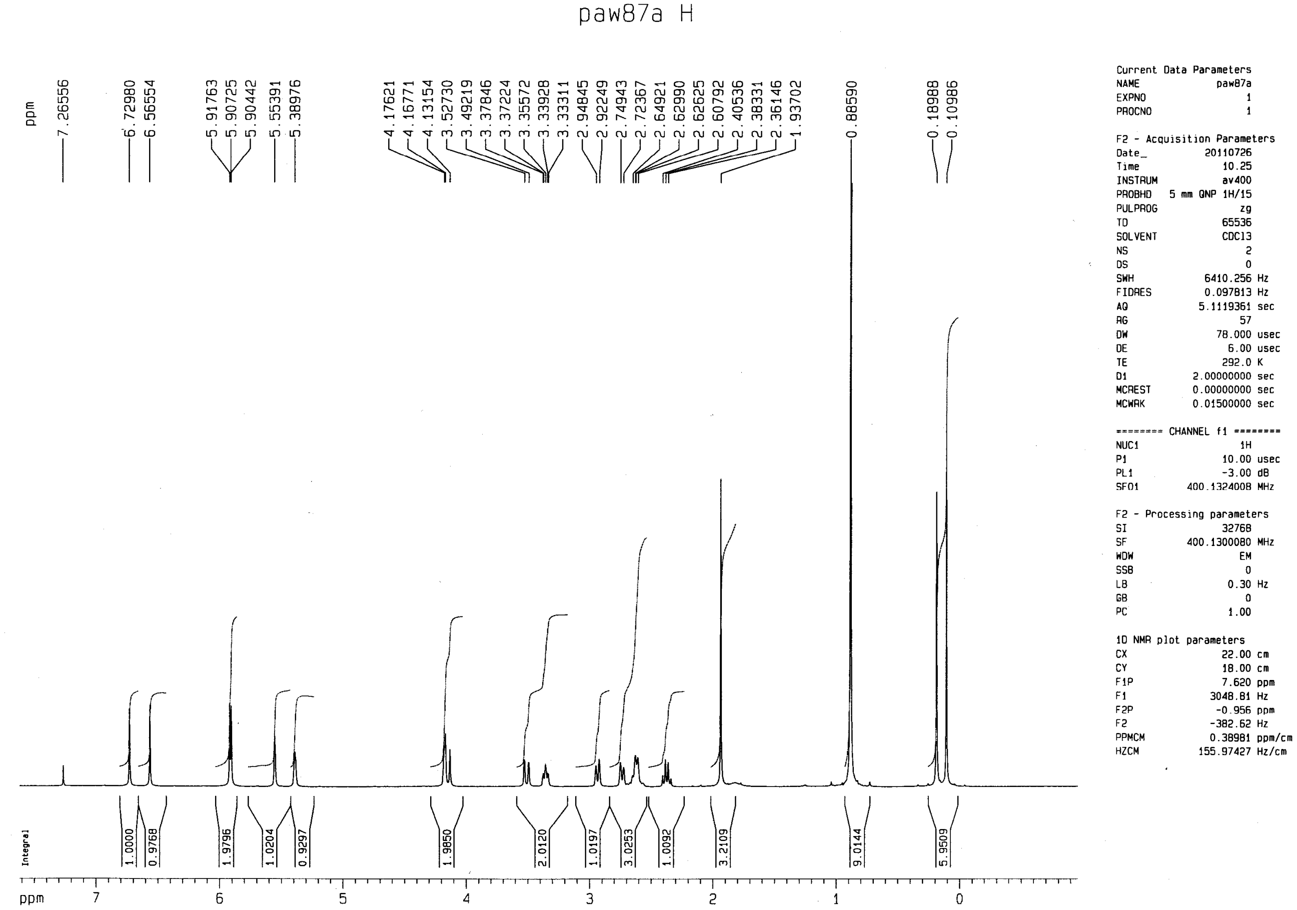

A11 ^{1}H-NMR spectrum of **7**

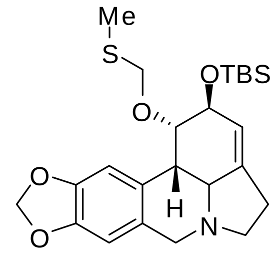

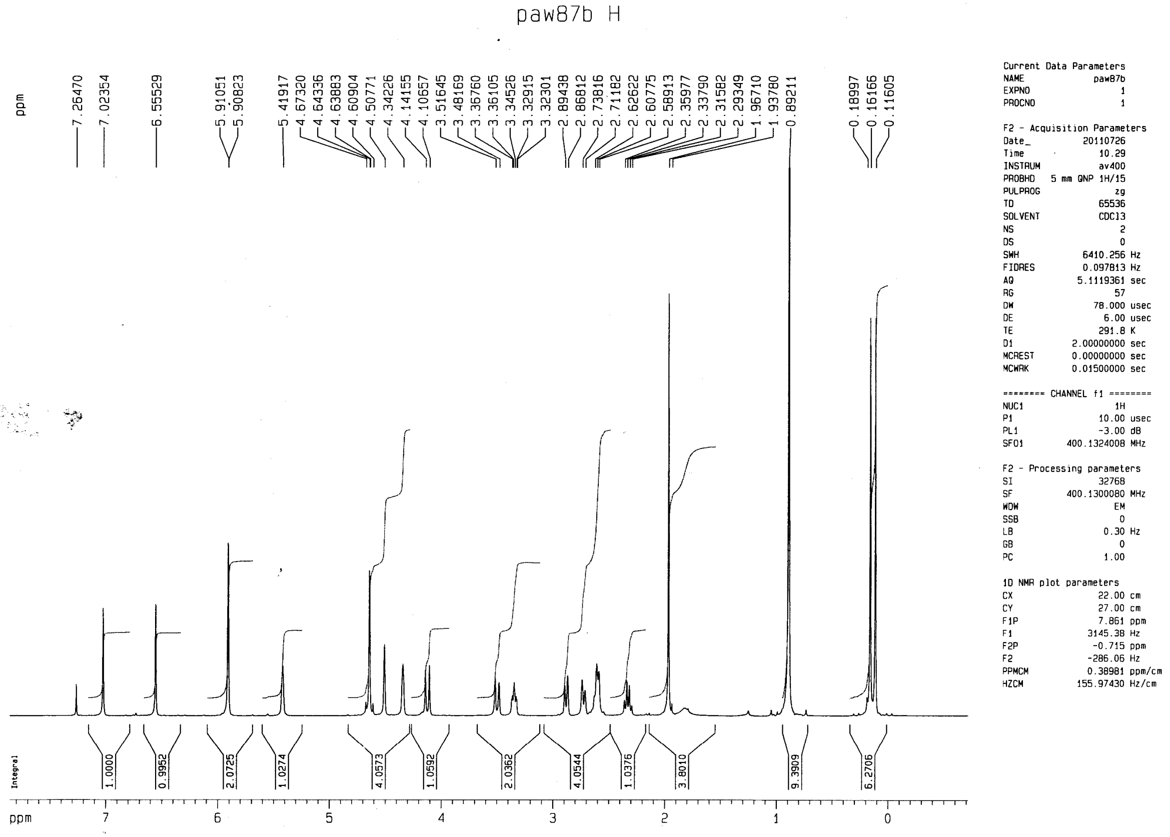

A12 ^{1}H-NMR spectrum of **8**

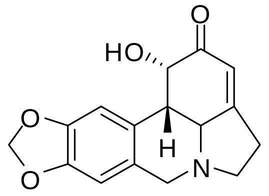

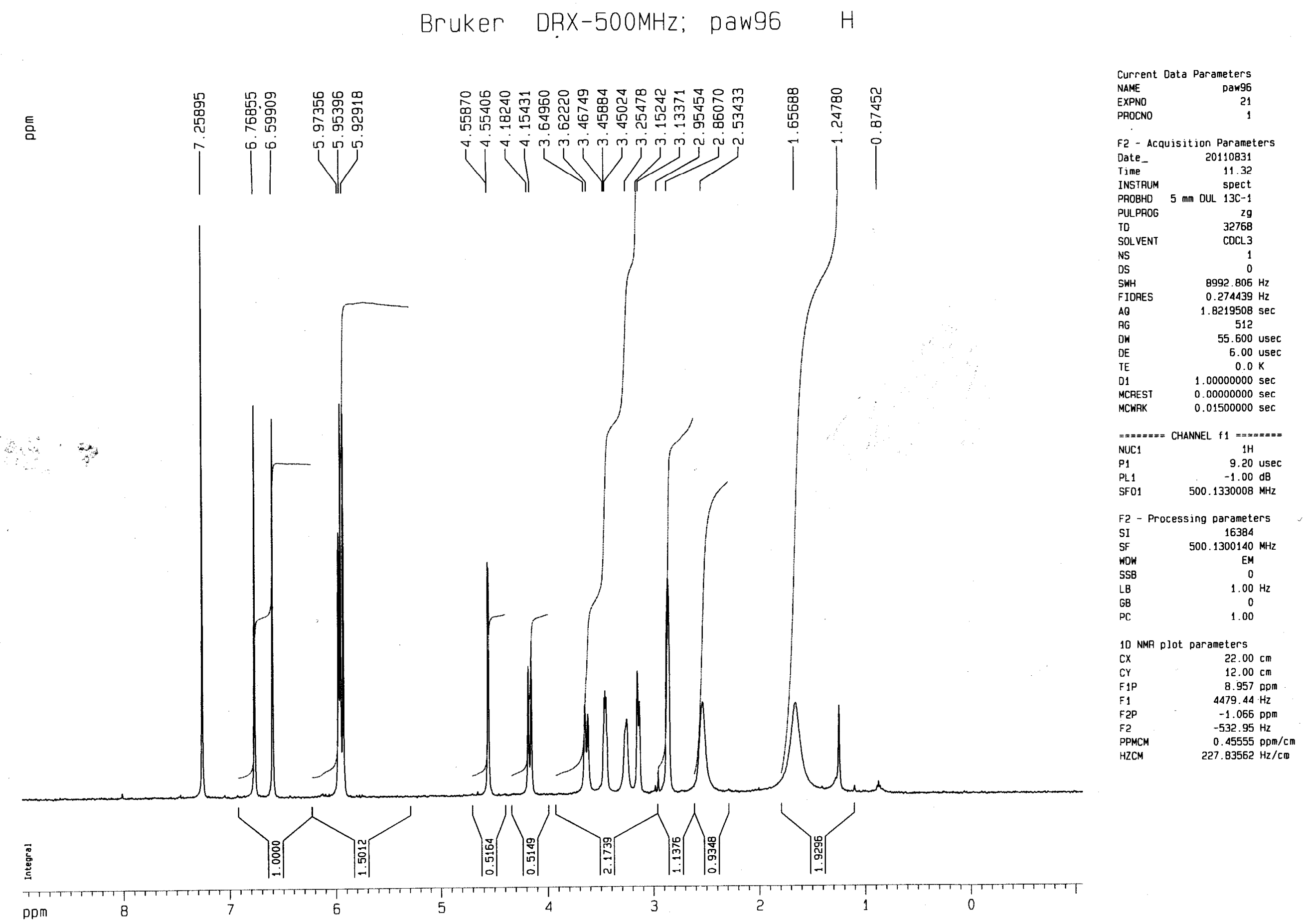

A13 ^{1}H-NMR spectrum of **9**

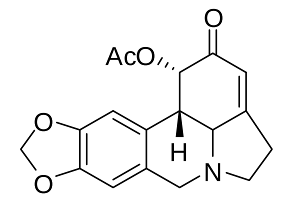

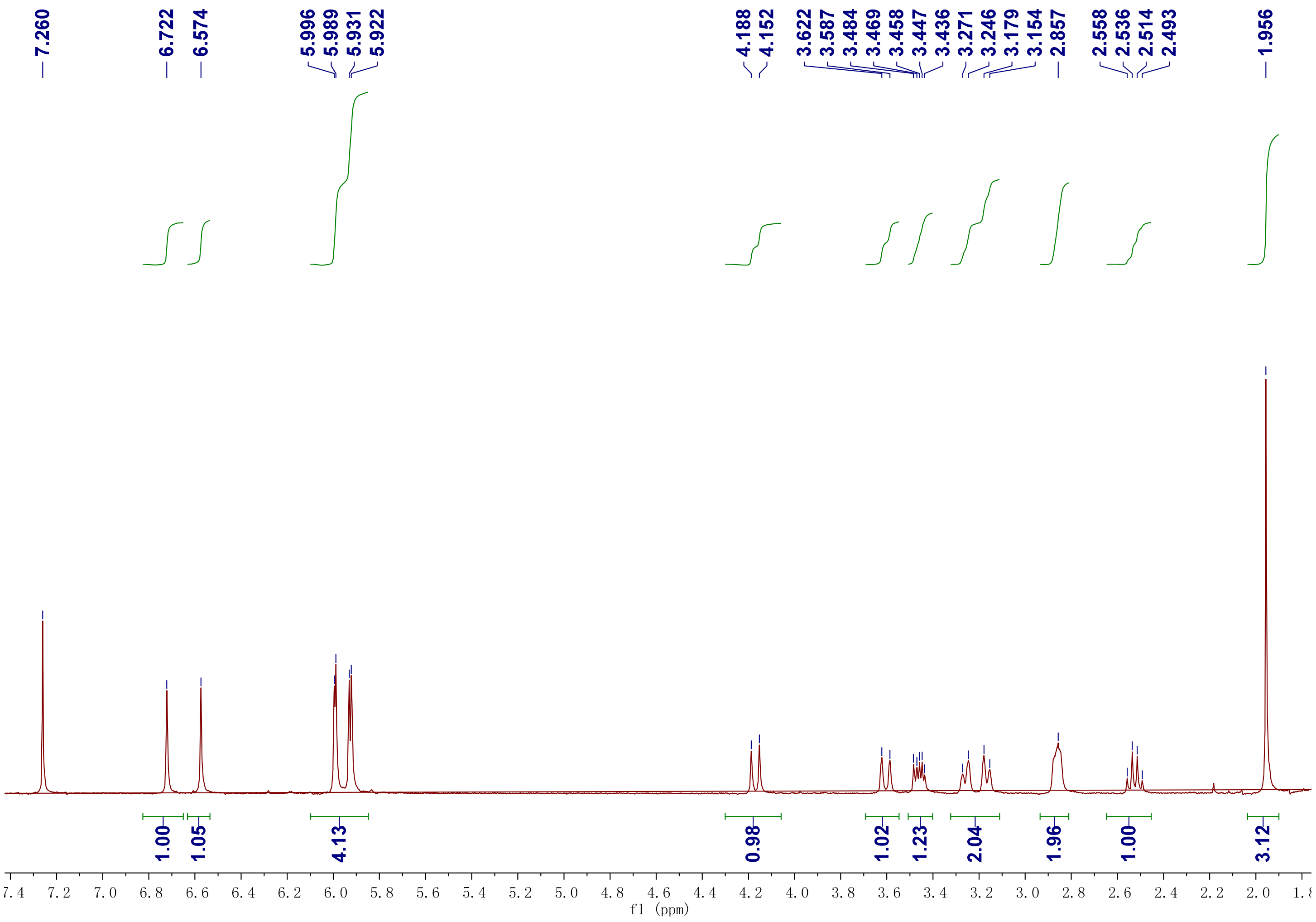

A14 ^{1}H-NMR spectrum of **10**

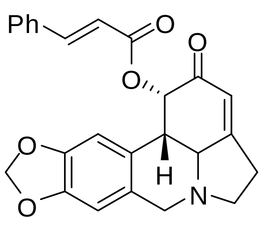

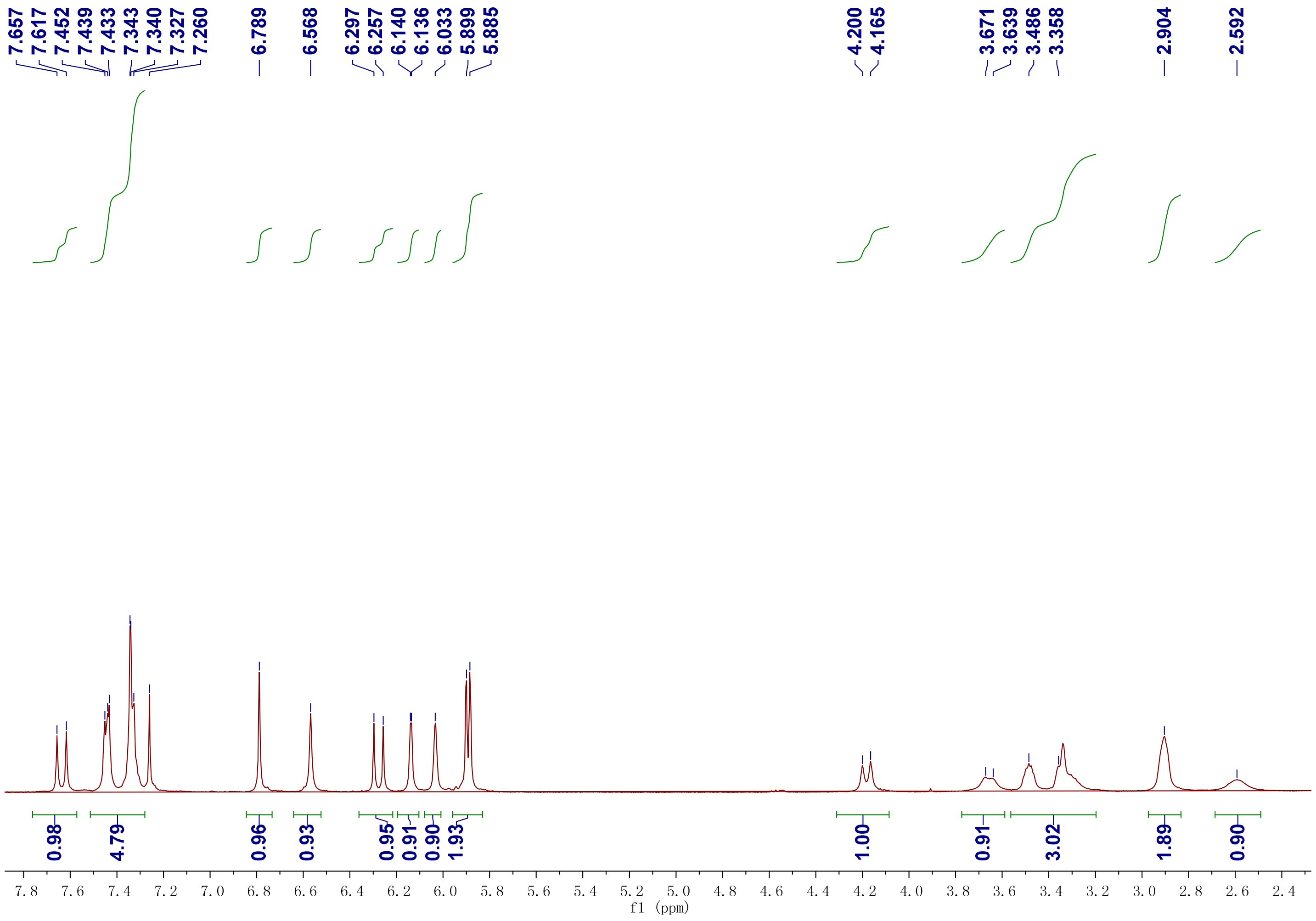

A15 ^{13}C-NMR spectrum of **10**

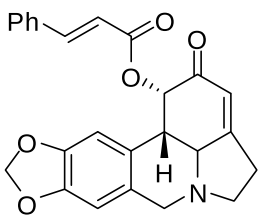

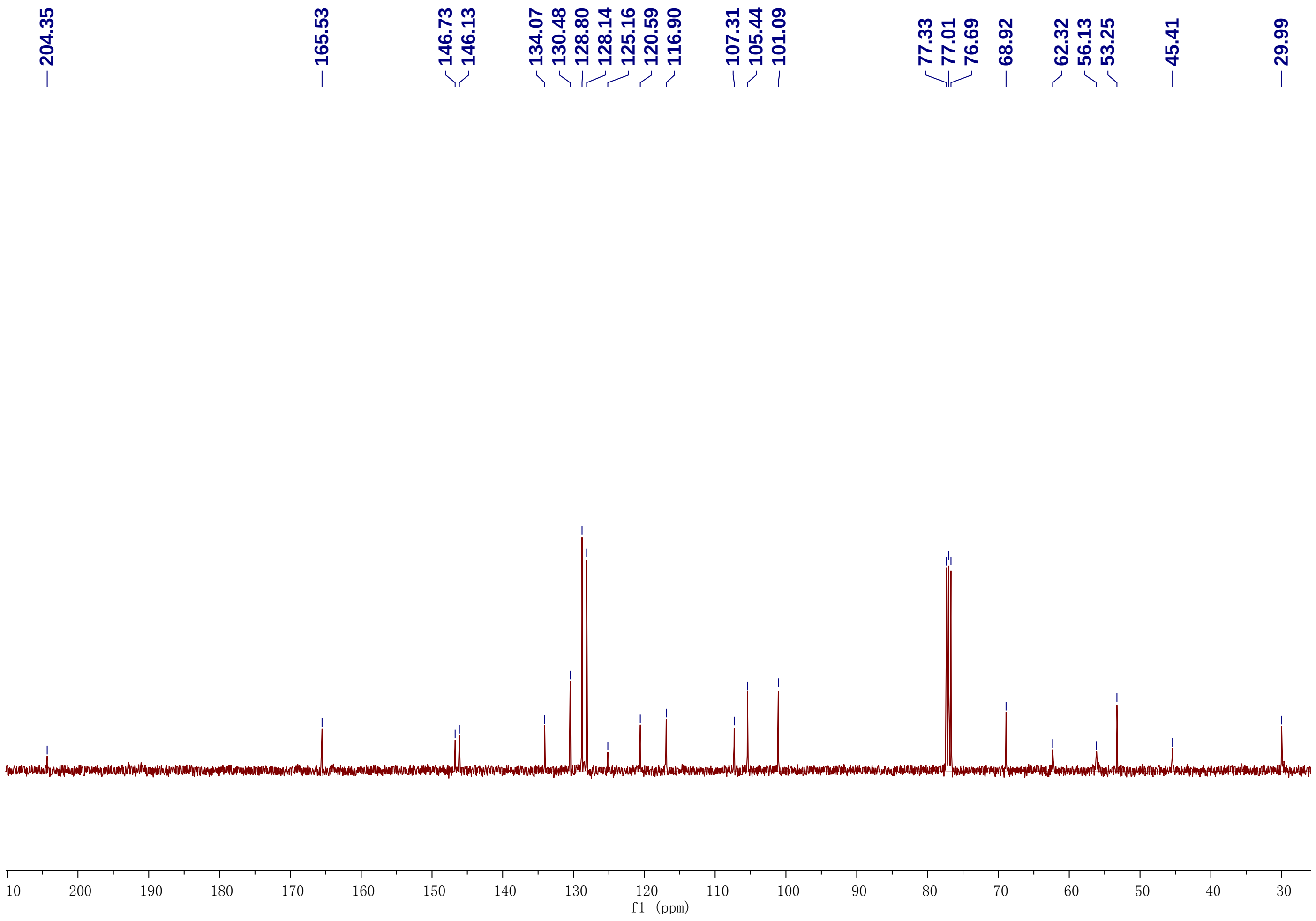

Supplement: Additional file 1 — NMR spectra of compounds 1–10. [file 1752-153X-6-96-S1.pdf]
